# Supplementary figures and images for: Significance of Lipid Fatty Acid Composition for Resistance to Winter Conditions in Asplenium scolopendrium
Source: Biology (Basel). 2022 Mar 25;11(4):507. doi: 10.3390/biology11040507 (PMC9024544; doi:10.3390/biology11040507)

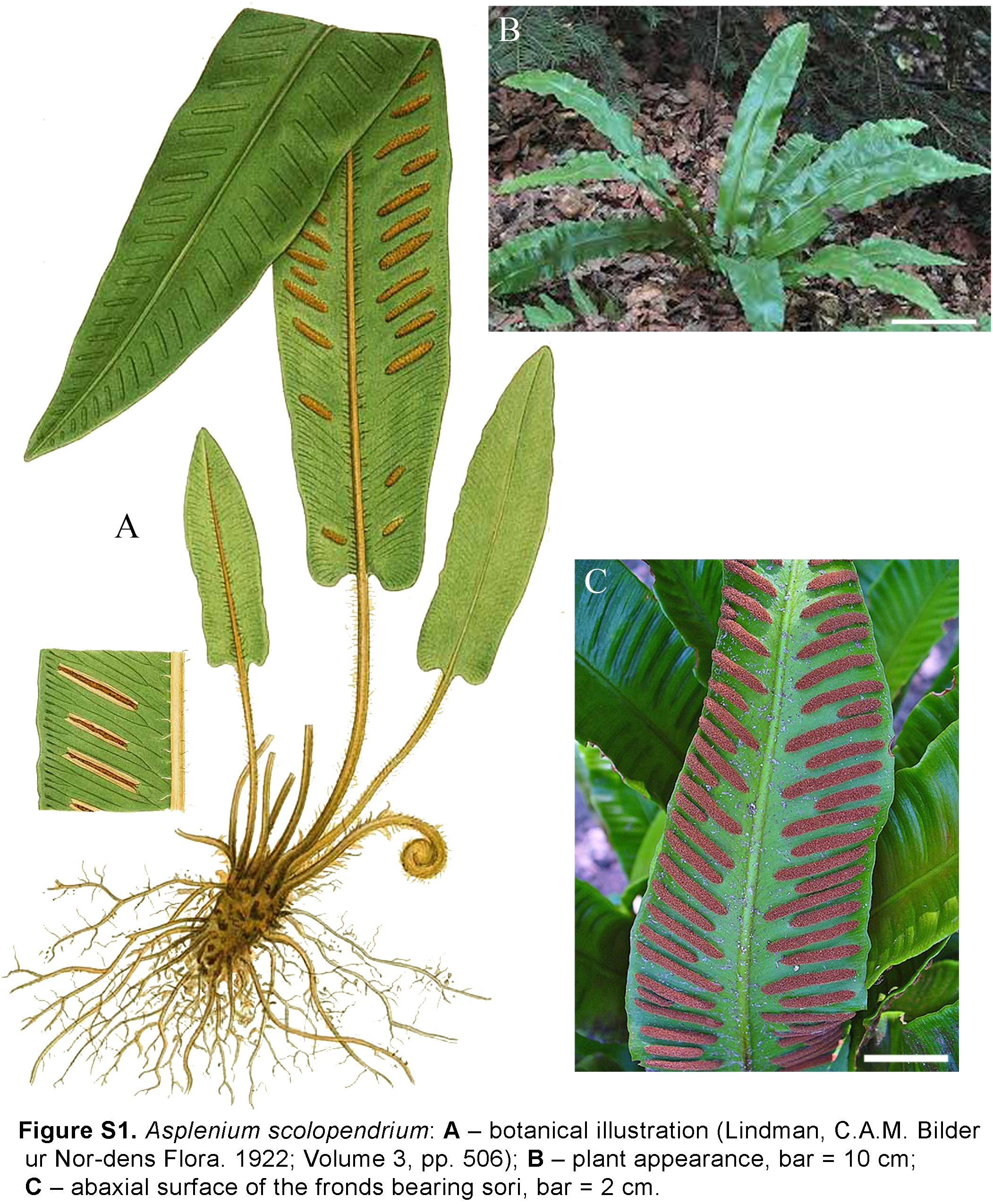

Supplement: Supplementary file 1 [file biology-11-00507-s001.zip › Figure S1.tif]

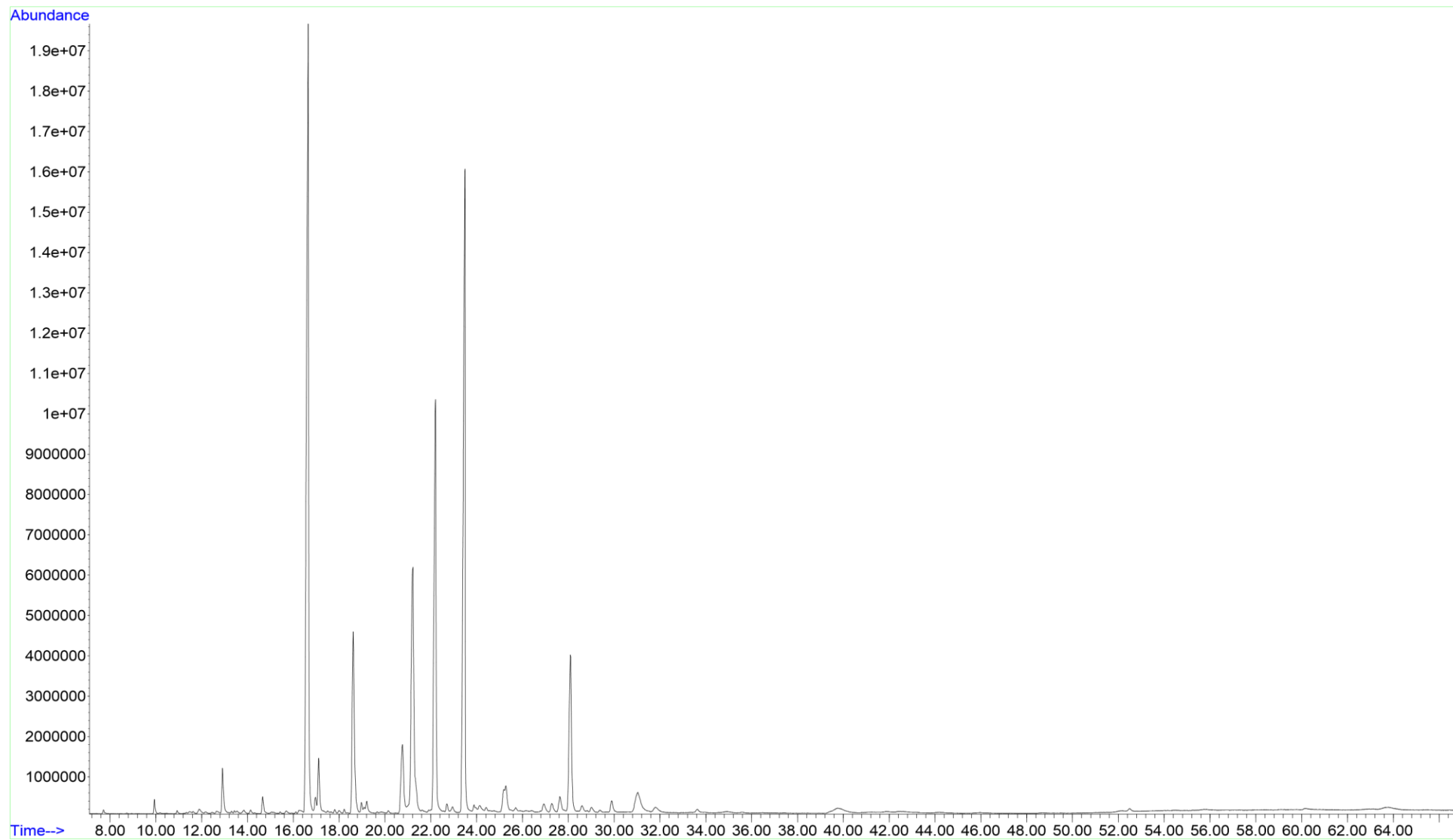

**Figure S2.** Chromatogram of *Asplenium scolopendrium* fatty acids.

Supplement: Supplementary file 1 [file biology-11-00507-s001.zip › Figure S2.pdf]
